# Epidemiological health assessment in primary healthcare in the State of Qatar- 2019

**DOI:** 10.5339/qmj.2021.57

**Published:** 2021-10-25

**Authors:** Mohamed Ghaith Al-Kuwari, Samya Ahmad Al-Abdulla, Maha Yousef Abdulla, Ahmad Haj Bakri, Azza Mustafa Mohammed, Mujeeb Chettiyam Kandy, Amanda Patterson, Jeyaram Illiayaraja Krishnan

**Affiliations:** ^1^Directorate of Strategy Planning and Health Intelligence, Primary Health Care Corporation, Doha, Qatar. E-mail: malkuwari@phcc.gov.qa; ^2^Directorate of Operations, Primary Health Care Corporation, Doha, Qatar.

**Keywords:** health needs, primary care, noncommunicable diseases, communicable diseases, Qatar

## Abstract

Background: In the public sector in Qatar, the Primary Health Care Corporation (PHCC) is the major provider of primary healthcare services to families. Therefore, the PHCC conducted the first epidemiological health assessment to understand the burden of diseases and their subsequent risk factors impacting its registered population, to design better services, implement it and allocate resources to respond to the population health needs.

Methods: A cross-sectional study design was adopted among all PHCC registered populations between September 1, 2018, and August 31, 2019. The study target population was all persons residing in Qatar aged 0+ years and registered at the 27 health centers affiliated with the PHCC; excluding patients with an expired residence permit on August 31, 2019, and craft male workers were provided their primary healthcare services at the Qatar Red Crescent health facilities. The data were extracted from patients’ electronic medical records (EMR).

Results: The burden of type 2 diabetes, hypertension, and dyslipidemia were the highest among the population of the central region at 13.9%, 15.7%, and 11.1%, respectively. Tobacco consumption among males was higher than females and ranged from 25.4% to 27.8%, with the highest rate in the northern region. Obesity rates ranged between 34.7% and 37.0% among the total population registered with the lowest rate in the central region, while 39.9% of females in the northern region had a body mass index above 30 kg/m^2^. Exclusive breastfeeding at 6 months was significantly lower than that at 4 months across all regions. Children in the northern region had the highest rate of overweight/obesity based on Z-scores. The western region population had the highest number of communicable diseases notifications.

Conclusion: Understanding the patterns of disease in the local population will enable the PHCC to plan a clear set of services that meet the population's health needs, which include tailored health education and promotion components.

## Introduction

Recent demographic and epidemiological trends point to populations living longer and with higher disease burden worldwide.^1^ Qatar's Second National Development Strategy (2017–2022) was expected to focus on healthcare, one of the eight priority sectors that will be integrated into sector projects, including the delivery of its newest National Health Strategy 2018–2022, which has a strong focus on primary care as the gateway to all other healthcare services.^2^ The nationally led programs, which aim to establish integrated care across the health sector will make great strides in ensuring patients with chronic disease and multiple noncommunicable diseases (NCDs) and will experience great coordination of care and better patient outcomes.^3^ Qatar has a dynamic population. In 2019, the total population was 2,666,938 inhabitants,^4^ 10% were Qataris and 90% were expatriates from various nationalities.^5^ The diversity of the expatriate population might be challenging for healthcare services planning. However, much of Qatar's strategic focus is on the Qatari population, as the long-term residents of the country, and where the greatest impact on health spending and future planning can be made and measured.

Furthermore, In Qatar, the highest cause of mortality in both men and women are diseases of the circulatory system.^3^ Cardiovascular disease is a major cause of health problems and deaths worldwide.^6^ It is also one of the most preventable causes of death linked to an unhealthy diet, lack of exercise, overweight, and smoking. When looking at NCDs collectively, 59.6% of males and 60.7% of females die every year from diseases that could either be prevented or better managed to allow patients to live longer and healthier lives.^3^

The high rates of NCDs also put considerable strain on secondary care services, causing a high rate of hospital attendances and admissions. According to the Centers for Diseases Control and Prevention in the United states the total estimated cost of diagnosed diabetes was 327 billion USD in medical cost and lost productivity in 2017.^7^ In Qatar, the prevalence of type 2 diabetes is projected to grow by 43% and consume one-third of Qatar's health expenditure by 2050.^8^

PHCC is the major public sector provider of primary care services to families in Qatar. PHCC operates in 27 health centers (March 2020) in three main health regions – northern, central, and western. The PHCC confirmed the region operationally to ensure easy allocation and management of health centers (Figure No.1). The registered active population in these health centers was 1,461,987 in February 2020, which is a 55% increase from 2015.^9^

### Aim

The epidemiological health assessment aims at providing a better understanding of PHCC targeted population health needs, risk factors, the prevalence of diseases, child health, and the incidence of communicable diseases across the three regions that PHCC health centers operate in.

## Methods

### Study design, population, and setting

A cross-sectional study design was implemented in the setting of the government primary healthcare centers affiliated with the PHCC. The target population was all persons residing in Qatar aged 0+ years and registered at the health centers affiliated with the PHCC, a total of 1,247,183 person

### Inclusion criteria

All the population registered at the PHCC health centers with a valid health card between September 1, 2018, and August 31, 2019, was included in the study.

### Exclusion criteria

All persons with an expired residence permit on August 31, 2018, and craft male workers that were registered with their primary healthcare services at the Qatari Red Crescent health centers were excluded.

### Data collection and data analysis

Data were extracted electronically from EMR covering the period between September 1, 2018, and August 31, 2019, per health center. Data extraction included all available information on the NCDs (diabetes excluding gestational, hypertension, dyslipidemia, depression, and anxiety); metabolic and behavioral risk factors (tobacco consumption, body mass index (BMI), Lipid Profile, and Glaucous profile); breastfeeding practices, and the z- score for weight-for-age captured during the well-baby clinic's visits; and the communicable diseases notifications.

Descriptive analysis was performed to calculate the proportion of diseases and conditions against their target population and p- values were calculated to track the total differences for the regions.

The burden of NCDs (diabetes, hypertension, dyslipidemia, depression, and anxiety) was assessed using the diagnosis from the field, excluding gestational diabetes and hypertension divided by the population at risk (population aged 18+). Metabolic and behavioral risk factors were assessed using the latest available value captured in the system between September 1, 2018, and August 31, 2019. Breastfeeding practices were evaluated based on the mothers’ responses to breastfeeding embedded questionnaire in their EMR at their visits to the well-baby clinics at the 4th and 6th months of the infant age. Children's obesity was assessed using the Z (standard) score developed by the World Health Organization to describe weight-for-age as the number of standard deviations above or below the reference mean/median value. The Z-score is gender independent, which permits comparison across populations and different age groups.^10^ The highest communicable diseases notifications were reported in their numbers, percentage of the total notifications, and incidence per 10,000 in their respective target population age group and nationality.

Data were presented for the three regions that the PHCC cluster is operating health centers under, to better understand the differences in the burden of diseases in the regions.

## Results

### Demography

The highest number of registered population on August 31, 2019, was in the central region with 531,568 persons corresponding to 42% of the PHCC total registered population. Children and young people aged from 0 to 18 years constituted 30% of the PHCC registered population across the three regions. Female percentages out of the total registered population were 45% in the western region and 48% in central and northern regions as shown in Tables 1 and 2. The male-to-female ratio was 1:1 among the three regions with a value higher in the western region at 1.2:1.

### Burden of NCDs

The registered population in the central region had the highest rates of NCDs. The burden of type 2 diabetes, excluding gestational diabetes, was the highest among the PHCC registered population in the central region at 13.9%, followed by the population registered in the northern and western regions at 12.6% and 11.9% respectively, with a P-value  < 0.001. The highest rate was among the male population in the central region at 16%. Hypertension rate was the highest among the registered population in the central region at 15.7% followed by population registered in the northern and western region at 12.5% and 11.8% respectively, with a P-value  < 0.001. Males in the central region had the highest rate at 18%. Dyslipidemia was the highest in the central region at 11.1% while it was the lowest in the western region at 7.8%. The male population registered in the central region had the highest rate at 12.6%. Depression rates ranged between 0.6% and 0.8% among the total population across the three regions with the highest rate identified among females registered in the central region at 1.1%. Co-morbidity ranged between 5.0% and 6.5% with the highest rate registered in the central region with a P-value  < 0.001. Anxiety ranged between 0.6% and 0.8% with the highest rate registered in the central and northern regions with a P-value  < 0.001 (Tables 1 and 2).

### NCDs risk factors prevalence

Tobacco consumption (Current smokers) among males ranged from 25.4% to 27.8%, with the highest rate in the northern region, as reported in Table 1.

Obesity rates ranged between 34.7% and 37.6% among the total population registered in the three regions with the lowest rate in the central region at 34.7% with a P-value  < 0.001. BMI values above 30 kg/m^2^ were higher in females than males with the highest rate among females in the northern region at 39.9% (Tables 1 and 2).

Glycated hemoglobin (HbA1c) over 6.5% was the highest among the population registered at the central region at 23.3% followed by the population registered at the northern and western region at 21.5% and 21.0% respectively, with a P-value  < 0.001. The highest rate was among the male population in the central region at 32.2% (Tables 1 and 2).

High cholesterol level - Cholesterol >6.2 mmol/L was highest among the population registered in the northern region at 7.4%. The rate was similar among the population registered in the central and western regions at 7% (Tables 1 and 2).

### Children overweight and obesity

The prevalence of overweight/obesity was consistently higher among children in the age groups 6–10 years old for both boys and girls than in the age group of 0–5 in all geographical regions. Additionally, the prevalence of overweight/obesity was consistently higher among boys than girls regardless of age in all geographical regions. In the northern region, overweight/obesity among younger children aged 0–5 was the highest at 7.2% compared with 6.6% and 6.4% in central and western regions, respectively, with a P-value  < 0.001. The same trend was observed among older children aged 6–10 where those residing in the northern region had the highest prevalence of overweight/obesity at 13.3% compared to 12.9% and 11.6% in central and western regions, respectively (Tables 1 and 2).

### Breastfeeding practices

Table 3 shows exclusive breastfeeding at 6 months was significantly lower than that at 4 months. This trend was observed in the three regions. In the central region, exclusive breastfeeding at 4 months among mothers was the highest at 30.2% compared to 26.0% and 25.7% in northern and western regions, respectively, with a P-value  < 0.001. Exclusive breastfeeding at 6 months ranged between 7.1% and 9.5% in the three regions with P-value  < 0.001.

### Communicable diseases notifications

The registered population in the western region had the highest number of communicable diseases notifications. Chickenpox notifications had the highest incidence rate at 94.6 children per 10,000 children aged between 5 and 9 years, while in the central and northern region the rate ranged from 47.2 and 48.8. Head lice notifications were the highest in the western region at 32.4 per 10,000 children aged between 4 and 5 years. Additionally, there was a higher incidence of MERS-CoV (Middle East respiratory syndrome) in the western region at 8.6 per 10,000 persons as illustrated in Table 4.

## Discussion

This paper presents the epidemiological analysis of the PHCC registered population stratified by regions for the first time to show the difference in needs. This analysis will allow health centers to plan their primary healthcare services in a decentralized approach.

The current study found that the demographic characteristics of the PHCC three operational regions (central, western, northern) were similar to the highest proportion of the registered population in the central region at 42%. Across the three regions, children and young people represented 30% of the total registered population, and the male-to-female ratio was 1:1. The latter demographic findings represent the population registered at the PHCC, which are mainly families and exclude the manual craft workers who are provided with their primary care services in Qatar Red Crescent health centers.^11^ In terms of urbanization, the three regions were operating in urban settings according to the Qatar population status report 2012 issued by the permanent population committee, which stated that urbanization pulled up to 100% in 2002 with 74% of the population concentrated in Doha and Al Rayan municipalities.^12^

Globally, NCDs kill 40 million people each year.^13^ Modifiable behavioral risk factors such as tobacco use, unhealthy diet, and physical inactivity, and harmful use of alcohol increases the risk of NCDs.^14^

In Qatar in 2012, the prevalence of diabetes among the Qatari population above 18 years old was 16.7% and the prevalence of high blood pressure among the same group was 33%.^15^ A recent estimate shows that the prevalence of diabetes mellitus will increase from 16.7% to at least 24.0% by 2050.^8^ However, in our study that included all populations registered at PHCC irrespective of their nationality, the prevalence of type 2 diabetes and hypertension was the highest in the central region at 13.9% and 15.7%, respectively. Thus, urbanization is associated with a higher awareness of the disease and better access to screening and diagnosis; bearing in mind that 75% of Qatar's population are residing in Doha^12^ in which the central region health centers cover most of the population there. The association between urbanization and NCDs was examined in various studies in low and middle-income countries in which evidence indicated that urbanization is associated with a higher prevalence of type 2 diabetes as well as increased blood pressure and cholesterol.^16^ However, this study needs to be explored more in future health - behaviors research within the culture and environment of Qatar.

A recent study conducted on the primary care population in Qatar had found that around 16.2% of the population that was studied had at least one of the following NCDs: type 2 diabetes, cardiovascular diseases, chronic obstructive pulmonary diseases, or cancer. Additionally, they reported that the burden of NCDs was higher among men than women.^17^ Our study supports those findings as it shows that the highest rates of type 2 diabetes, hypertension, and dyslipidemia were among the male population in the central region.

Obesity in Qatar was reported at 40% among Qataris aged 18+ years according to STEPwise 2012.^15^ However, in this study, obesity rates ranged between 34.7% and 37.0% among the total population registered in the three regions. Females had the highest rate in the northern region at 39.9%. The latter finding indicates that obesity is high among the populations registered at PHCC irrespective of their nationality. However, the differences in rates between the regions can be attributed to ethnicity, socio-economic, and education factors that need to be more examined in a special type of social behavioral study. According to Stunkard's study, socio-economic status and education are contributors to obesity.^18^

An additional study conducted in 2018 among students in Qatar aged between 5 and 19 years, reported an overall overweight and obesity prevalence of 44.8% and 40.4% among males and females, respectively. Additionally, they found that among students “males had 1.48 times higher odds of having obesity than females.”^19^ The latter study noticed the same trend as in our study, which demonstrated that among children aged 0-10 the prevalence of overweight/obesity was consistently higher among boys than girls in all regions. We also found that the rate of obesity increases with the increase of age among those children.

Interestingly, NCDs control was identified as perceived needs by the community in Qatar. A recent qualitative study conducted in 2019 found that individuals registered in PHCC have identified diabetes, hypertension, obesity, and physical inactivity as major health problems that need to be prioritized in the curative services and invest in more community-based preventive interventions.^20^

Tobacco consumption among males in our study ranged from 25.4% to 27.8%, which is higher than the overall smoking prevalence of 17.9% among males reported in the global adult tobacco survey in 2013.^21^ In a recent study conducted in Qatar in 2018 tobacco consumption among Qataris, males were at 20.6%.^22^ The latter findings can be attributed to ask about smoking status as part of the vital signs reporting in each visit. However, there is a need for further studies to understand the reason for the high smoking rates despite all efforts put into tobacco control including legislative measures, taxation, community awareness, and providing smoking cessation services since 2002.^23^

In terms of communicable diseases notifications, the western region population had the highest number with chickenpox at 94.6 children per 10,000 children aged between 5 and 9 years. Although “Qatar was the first in the Middle East region to introduce the chickenpox vaccine in 2002,”^24^ we still see varicella infections. Chickenpox is more prevalent among school-age children compared to younger children. Historical vaccination data collected at the primary care level in Qatar suggests that these children have missed their second vaccine dose, which boosts the immunity of the children and enhances the protective effect of the vaccine. Additionally, the long period between the first dose administered at 12 months and the second one administered between 4 and 6 years is a contributing factor for vaccine default. Another reason can be that the varicella vaccine is not routine in many countries, including countries of origin for the vast majority of the non-Qatari population e.g. Middle East and Asian countries.^25^ It's important to have continuous surveillance to examine the need for catchup immunizations especially for populations at risk that have interrupted or delayed vaccinations.^24^ The western region has more MERS-CoV notifications which are related to the presence of the camel farms in that region for racing and breeding. Various studies showed a high prevalence of MERS-CoV antibodies in dromedary camels in the Arabian Peninsula.^26^ According to a study conducted in 2019, the role of camels in the transmission of MERS-CoV was documented in Qatar.^27^

Breastfeeding practices remain low in Qatar; the available data from 2012 showed that only 29.3% of infants aged 0-5 months were exclusively breastfed.^28^ Another recent study conducted in 2017 reported that the total number of children being exclusively breastfed (12%) is significantly lower than the international rate (37%).^29^ And, it identified some barriers to continued breastfeeding, which includes the perception of breastfeeding as painful, body image, the difficulty of breastfeeding in public, or at work.^28^ The breastfeeding rate at 6 months was the lowest among Qatari women in a study conducted in 2019.^30^ In this assessment, exclusive breastfeeding at 6 months was significantly lower than that at 4 months. This trend was observed in the three regions. In the central region, however, exclusive breastfeeding at 4 months among mothers was the highest at 30.7%. The latter results can be attributed to urbanization and the increased percentage of women entering the labor market. According to the World Bank report 2021, the percentage of women in Qatar aged 15+ years participating in the labor force in 2019 rose from 48% in 2009 to 57% in 2019.^31^ These findings highlight the crucial need for community-based programs that encourage exclusive and continued breastfeeding. Health educators and other healthcare providers can work with mothers to support them, improve their breastfeeding skills, and engage other family members to provide additional support for them. Furthermore, this study needs to be communicated with policymakers to advocate for breastfeeding-friendly workplaces and longer maternity leave.^29^ Additionally, the Qatar National Health Strategy 2018-2022 has a specific national target to increase the level of exclusive breastfeeding of children at 6 months of age by 15% under the children's health priority population.^2^

### Strengths and limitations

The main limitation of this study is that the data were collected retrospectively through the patients’ EMR with the latest available value captured in the system during the defined period. This limitation can subject the study to incomplete data for certain variables, namely, the social and behavioral variables. An additional limitation was that the studied subjects were the population registered at the PHCC health centers, excluding the manual craft male workers who are registered in Qatar Red Crescent health centers.

The strength of the epidemiological health assessment derives from including all the population registered at the PHCC and stratified by region for the first time.

## Conclusion

The findings of this study illustrated that the prevalence of NCDs and their subsequent risk factors and communicable diseases notifications varied across the three regions that the primary healthcare centers are geographically distributed in Qatar. Hence, understanding the patterns of disease in the local population will enable the PHCC to provide a clear set of objectives to work toward improving the health conditions of its target population. The importance of assessing health needs rather than reacting to health demands is widely recognized. The above findings will be integrated into the planning and commissioning of primary healthcare services across the country. Recommendations include the increased use of integrated NCDs model in the primary healthcare services to provide early NCDs treatment, self-care, and prevention. It also recommended developing plans to educate the population regarding the adverse health effects of tobacco consumption and implementing additional measures to encourage healthy eating habits and exercise; to reduce the prevalence of type 2 diabetes, obesity, and hypertension. Additionally, the health of children regarding breastfeeding and obesity prevention needs to be considered as part of the services.

## Declarations

### Ethics approval

The epidemiological health assessment was approved by the Primary Health Care Corporation Corporate Strategy Implementation group at Primary Health Care Corporation in April 2019 as planning tool under the strategic initiative CSP 4.1.1. The full report was released in October 2020 for the public according to the approval of the Primary Health Care Corporation managing director to comply with the strategy and service planning requirements of Accreditation Canada International.

### Conflict of interests

The corresponding authors and all the co-authors of this paper declare that they have no conflict of interest.

### Data availability statement

The datasets generated during and/or analyzed during the epidemiological health assessment are available from the corresponding author on reasonable request.

### Funding

This research received no external funding.

### Authors contributions

All the authors contributed to the development of the study protocol, AHB and AM conducted the statistical analysis and produced the results, and all the authors reviewed and feedbacked on the results and contributed to the manuscript development.

### Acknowledgment

We would like to extend our gratitude for the Business Health Intelligence department at the Primary Health Care Corporation in Qatar for providing all the needed support for the data extraction.

## Figures and Tables

**Figure 1.**
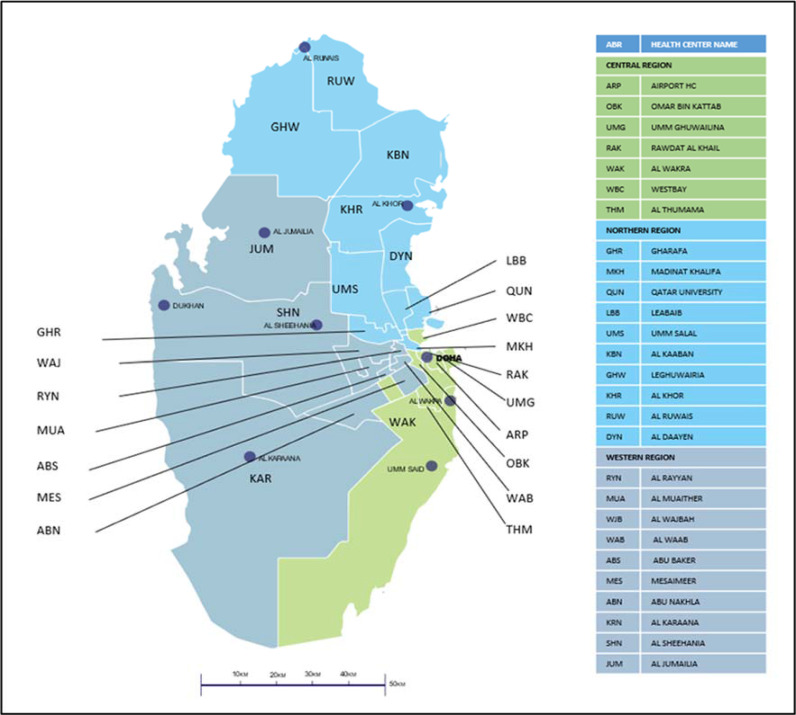
Qatar Primary Health Care Corporation health centers catchment areas

**Table 1:** Primary Health Care Corporation epidemiological health assessment by region

**Primary Health Care Corporation Regions**	**Central**		**Western**		**Northern**		

**Demography**							

Age-group	Total		Total		Total		P value

	n	**%**	n	**%**	n	**%**

0-18 years old	158,166	**29.8**	109,798	**32.7**	118,088	**31.1**	< 0.001

19+ years old	373,402	**70.2**	226,437	**67.3**	261,292	**68.9**	< 0.001

Total	531,568	**100**	336,235	**100**	379,380	**100**	

**Burden of NCDs**							

Diabetes	51,843	**13.9**	26,993	**11.9**	32,969	**12.6**	< 0.001

Hypertension	58,714	**15.7**	26,758	**11.8**	32,692	**12.5**	< 0.001

Dyslipidemia	41,329	**11.1**	17,579	**7.8**	23,143	**8.9**	< 0.001

Depression	3,137	**0.8**	1,291	**0.6**	1,845	**0.7**	< 0.001

Anxiety	2,965	**0.8**	1,441	**0.6**	2,036	**0.8**	< 0.001

Co-morbidity	24,447	**6.5**	11,258	**5.5**	13,972	**5.3**	< 0.001

**Behavioral risk factors**							

	Total (N = 199,754)		Total (N = 106,147)		Total (N = 98,204)		

	n	%	n	%	n	%	

Current smoker	27,454	**13.7**	14846	**14**	13618	**13.9**	0.173

**Metabolic risk factors**							

	Total (N = 179,249)		Total (N = 103,222)		Total (N = 116,03)		

	n	%	n	%	n	%	

Overweight - BMI between 25-30 kg/m^2^	66,936	**37.6**	36,966	**35.8**	41,523	**35.8**	< 0.001

Obese - BMI 30+ kg/m^2^	61,794	**34.7**	38,162	**37**	42,472	**36.6**	< 0.001

	Total (N = 11,3163)		Total (N = 64,876)		Total (N = 75,056)		

	n	%	n	%	n	%	

Glycated hemoglobin (HbA1c) - 5.7%-6.4%	29,105	**25.7**	16,154	**24.9**	18,743	**25.0**	< 0.001

Glycated hemoglobin (HbA1c) - 6.5% or higher	26,410	**23.3**	13,610	**21.0**	16,135	**21.5**	< 0.001

	Total (N = 112,726)		Total (N = 61,717)		Total (N = 72,115)		

	n	%	n	%	n	%	

High Cholesterol level – Cholesterol >6.2 mmol/L	7,926	**7**	4,286	**6.9**	5,303	**7.4**	0.006

**Children overweight and obesity (0-5) years**							

	Total (N = 121,068)		Total (N = 141,300)		Total (N = 102,863)		

	n	%	n	%	n	%	

Overweight/Obesity	8,004	**6.6**	9,089	**6.4**	7,376	**7.2**	< 0.001

**Children overweight and obesity (6-10) years**							

	Total (N = 46,189)		Total (N = 66,955)		Total (N = 43,757)		

	n	%	n	%	n	%	

Overweight/Obesity	5,967	**12.9**	7,784	**11.6**	5,794	**13.3**	< 0.001


**Table 2:** Primary Health Care Corporation epidemiological health assessment by region and sex

Primary Health Care Corporation Regions	**Central**				**Western**				**Northern**			

**Demography**												

Age-group	Female		Male		Female		Male		Female		Male	

	n	**%**	n	**%**	n	**%**	n	**%**	n	**%**	n	**%**

0-18 years old	77,213	**29.7**	80,953	**29.8**	53,567	**34.9**	56,231	**30.8**	57,595	**31.1**	60,493	**31.2**

19+ years old	182,572	**70.3**	190,830	**70.2**	99,812	**65.1**	126,625	**69.2**	127,781	**68.9**	133,511	**68.8**

Total	259,785	**100**	271,783	**100**	153,379	**100**	182,856	**100**	185,376	**100**	194,004	**100**

**Burden of NCDs**												

Diabetes	21,399	**11.7**	30,444	**16**	12,641	**12.7**	14,352	**11.3**	15,396	**12.0**	17,573	**13.2**

Hypertension	24,429	**13.4**	34,285	**18**	11,776	**11.8**	14,982	**11.8**	15,209	**11.9**	17,483	**13.1**

Dyslipidemia	17,300	**9.5**	24,029	**12.6**	7,484	**7.5**	10,095	**8.0**	10,670	**8.4**	12,473	**9.3**

Depression	1,951	**1.1**	1,186	**0.6**	832	**0.8**	459	**0.4**	1248	**1.0**	597	**0.4**

Anxiety	1,663	**0.9**	1,302	**0.7**	776	**0.8**	665	**0.5**	1239	**1.0**	797	**0.6**

Co-morbidity	8,779	**4.8**	15,668	**8.2**	4,162	**4.2**	7,096	**5.6**	5,663	**4.4**	8,309	**6.2**

**Behavioral risk factors**												

	F (N = 103,859)		M (N = 95,895)		F (N = 52,502)		M (N = 53,645)		F (N = 53,190)		M (N = 45,014)	

	n	%	n	%	n	%	n	%	n	%	n	%

Current smoker	2,732	**2.6**	24,722	**25.8**	1,201	**2.3**	13,645	**25.4**	1,105	**2.1**	12,513	**27.8**

**Metabolic risk factors**												

	F (N = 97,500)		M(N = 80,749)		F (N = 59,075)		M (N = 44,147)		F (N = 66,295)		M (N = 49,738)	

	n	%	n	%	n	%	n	%	n	%	n	%

Overweight - BMI between 25-30 kg/m^2^	32,979	**33.8**	33,957	**42.1**	19,233	**32.6**	17,733	**40.2**	21,253	**32.1**	20,270	**40.8**

Obese - BMI 30+ kg/m^2^	35,964	**36.9**	25,830	**32**	23,525	**39.8**	14,637	**33.2**	26,433	**39.9**	16,039	**32.2**

	F (N = 60,171)		M (N = 52,992)		F (N = 35,937)		M (N = 28,939)		F (N = 42,054)		M (N = 33,002)	

	n	%	n	%	n	%	n	%	n	%	n	%

Glycated hemoglobin (HbA1c) - 5.7%-6.4%	14,491	**24.1**	14,614	**27.6**	8,230	**22.9**	7,924	**27.4**	9,702	**23.1**	9,041	**27.4**

Glycated hemoglobin (HbA1c) - 6.5% or higher	9,346	**15.5**	17,064	**32.2**	5,229	**14.6**	8,381	**29.0**	6,534	**15.5**	9,601	**29.1**

	F (N = 55,514)		M (N = 57,212)		F (N = 31,646)		M (N = 30,071)		F (N = 37,967)		M (N = 34,148)	

	n	%	n	%	n	%	n	%	n	%	n	%

High Cholesterol level – Cholesterol >6.2 mmol/L	3,703	**6.7**	4,223	**7.4**	1,915	**6.1**	2,371	**7.9**	2,518	**6.6**	2,785	**8.2**

Children overweight and obesity (0-5) years												

	Girls (N = 58,465)		Boys (N = 62,603)		Girls (N = 68,157)		Boys (N = 73,143)		Girls (N = 49,728)		Boys (N = 53,135)	

	n	%	n	%	n	%	n	%	n	%	n	%

Overweight/Obesity	3,497	**5.9**	4,507	**7.2**	3,957	**5.8**	5132	**7.0**	3,178	**6.4**	4,198	**7.9**

**Children overweight and obesity (6-10) years**												

	Girls (N = 22,897)		Boys (N = 23,292)		Girls (N = 32,285)		Boys (N = 34,670)		Girls (N = 21,661)		Boys (N = 22,096)	

	n	%	n	%	n	%	n	%	n	%	n	%

Overweight/Obesity	2,571	**11.2**	3,396	**14.6**	3,331	**10.3**	4,453	**12.8**	2,578	**11.9**	3,216	**14.5**


**Table 3:** Exclusive breastfeeding practices at 4 and 6 months by region

**Primary Health Care Corporation Regions**	**Central**		**Western**		**Northern**		P value

							

	(N = 2,815)		(N = 2,303)		(N = 1,419)		

	n	%	n	%	n	%	

Exclusive breast feeding at 4 months	850	**30.2**	591	**25.7**	369	**26.0**	< 0.001

	(N = 2,906)		(N = 2,228)		(N = 1,735)		

	n	%	n	%	n	%	

Exclusive breast feeding at 6 months	207	**7.1**	212	**9.5**	151	**8.7**	< 0.001


**Table 4:** Communicable diseases notification incidence by region

CD Notification order by reported numbers	1		2		3		4		5	

**Primary Health Care Corporation Regions**	n	Incidence per 10,000	n	Incidence per 10,000	n	Incidence per 10,000	n	Incidence per 10,000	n	Incidence per 10,000

Central	Flu-like illness		Chicken pox		Scabies		Shingles		Head lice	

	806	**15.2**	238	**47.2**	199	**3.7**	293	**38.2**	99	**11.0**

Western	Chicken pox		Head lice		Influenza like illness		MERS - Middle East respiratory syndrome		Scabies	

	356	**94.6**	211	**32.8**	202	**6.0**	191	**8.6**	189	**5.6**

Northern	Influenza like illness		Shingles		Chicken pox		Scabies		Hand foot and mouth disease	

	288	**7.6**	224	**49.9**	185	**48.8**	140	**3.7**	128	**18.8**


## References

[1] Hay SI, Abajobir AA, Abate KH, Abbafati C, Abbas KM, Abd-Allah F, et al. Global, regional, and national disability-adjusted life-years (DALYs) for 333 diseases and injuries and healthy life expectancy (HALE) for 195 countries and territories, 1990–2016: a systematic analysis for the Global Burden of Disease Study. *Lancet.* 2016;390(10100):1260–344 10.1016/S0140-6736(17)32130-X PMC5605707 28919118

[2] Ministry of Public Health. National health strategy 2018-2022. Doha. Ministry of Public Health .2018. [cited 2021 Mar 3]. Available from: https://www.moph.gov.qa/Admin/Lists/PublicationsAttachments/Attachments/54/NHS.pdf

[3] Ministry of Public Health. Qatar Health Report 2014–2016[Internet]. Doha. Ministry of Public Health. 2017. Qatar.[cited 2021 Mar 2]. Available from: https://www.moph.gov.qa/english/Search/Pages/results.aspx?k = Qatar%20health%20report

[4] Planning and Statistics Authority. Key indicators [Internet]. Doha. Planning and Statistic Authority. 2020. [cited 2021 Mar 2] Available from: https://www.psa.gov.qa/en/statistics1/StatisticsSite/Pages/KeyIndicators.aspx

[5] Priya Dsouza Communications. Population of Qatar by nationality – 2019 report [Internet]. Doha. Priya Dsouza Communications. 2020. [cited 2021 Mar 3]. Available from: http://priyadsouza.com/population-of-qatar-by-nationality-in-2017/

[6] Mc Namara K, Alzubaidi H, Jackson JK. Cardiovascular disease as a leading cause of death: how are pharmacists getting involved? Integr Pharm Res Pract. 2019;8:1–11. doi: 10.2147/iprp.s133088 PMC6366352 30788283

[7] Centers for Diseases Control and Prevention. Health and Economics Costs of Chronic Diseases [Internet]. Atlanta. Centers for Diseases Control and Prevention. 2021. [cited 2021 Oct 10]. Available from: https://www.cdc.gov/chronicdisease/about/costs/index.htm

[8] Awad SF, O'Flaherty M, Critchley J, Abu-Raddad LJ. Forecasting the burden of type 2 diabetes mellitus in Qatar to 2050: a novel modeling approach. Diabetes Res Clin Pract. 2018;137:100–108. doi: 10.1016/j.diabres.2017.11.015 29175341

[9] Primary Health Care Corporation. Primary Health Care Corporation corporate strategic plan 2019 – 2023 [Internet]. Doha. Primary Health Care Corporation. 2019. [cited 2021 Mar 10]. Available from: https://Primary Health Care Corporation.qa/portal_new/admin/images/documents/2019/Corporate%20Strategic%20Plan%202019-2023%20English.pdf

[10] World Health Organization. Training course on child growth assessment [Internet]. Geneva, WHO; 2008. [cited 2021 Aug 28] Available from: https://www.who.int/childgrowth/training/module_c_interpreting_indicators.pdf

[11] Qatar Red Crescent. Workers Health Centers. Doha. Qatar Red Crescent. 2021.[cited 2021 Jul 2] Available on: https://www.qrcs.org.qa/en/Pages/HealthCenters.aspx

[12] Ministry of Development and Statistics. Qatar population status 2012. Permanent Population Committee, Doha; 2012. [cited 2021 Jul 2]. Available from https://www.ppc.gov.qa/Admin/QatarPopulationReport/PPC_Qatar_Population_Status_2012_EN.pdf#search = urban

[13] World Health Organization. Noncommunicable diseases [Internet]. Geneva. World Health Organization. 2021 [cited 2021 Mar 2]. Available from http://www.who.int/mediacentre/factsheets/fs355/en/

[14] GBD 2015 Risk Factors Collaborators. Global, regional, and national comparative risk assessment of 79 behavioural, environmental and occupational, and metabolic risks or clusters of risks, 1990–2015: a systematic analysis for the Global Burden of Disease Study 2015. *Lancet.* 2016. 388(10053):1659–1724 10.1016/S0140-6736(16)31679-8 PMC5388856 27733284

[15] Haj Bakri A, Al-Thani A. Chronic disease risk factor surveillance: Qatar STEPS report 2012. Doha. The Supreme Council of Health. 2013

[16] Koch M. Urbanisation, inequality, and non-communicable disease risk. Lancet Diabetes Endocrinol. 2017;5(5):313. doi: 10.1016/s2213-8587(17)30116-x 28395876

[17] Syed MA, Alnuaimi AS, Zainel AJ, Hamda AA. Prevalence of non-communicable diseases by age, gender and nationality in publicly funded primary care settings in Qatar. *BMJ Nutr Prev Health.* 2019;2(1):20 10.1136/bmjnph-2018-000014 PMC7678476 33235953

[18] Pavela G, Lewis DW, Locher J, Allison DB. Socioeconomic status, risk of obesity, and the importance of Albert J. Stunkard. Curr Obes Rep. 2016;5(1):132–139. doi: 10.1007/s13679-015-0185-4 PMC4798886 26746415

[19] Al-Thani M, Al-Thani A, Alyafei S, Al-Chetachi W, Khalifa SE, Ahmed A, et al. The prevalence and characteristics of overweight and obesity among students in Qatar. Public Health. 2018;160:143–149. doi: 10.1016/j.puhe.03.020 29704956

[20] Al-Kuwari MG, Al Abdulla S, Abdulla M, Mohammed AM, Bakri AH, Shaikhan F, Buhaddoud H. Qualitative focus group study examining perceptions of the community's important health issues, health care needs and perceived barriers to access among Arabic speaking primary care clients in the State of Qatar. J Multidiscip Healthc. 2021;14:961–971. doi: 10.2147/jmdh.s288194 PMC8090979 33953565

[21] Supreme Council of Health. Global adult tobacco survey [Internet]. Supreme Council of Health. Doha; 2013. [cited 2021 Jul 4] Available from https://www.psa.gov.qa/en/statistics/Surveys/GATS-BOOK.pdf

[22] AlMulla A, Mamtani R, Cheema S, Maisonneuve P, Abdullah BaSuhai J, Mahmoud G, et al. Epidemiology of tobacco use in Qatar: prevalence and its associated factors. PLoS ONE. 2021;16(4):e0250065. doi: 10.1371/journal.pone.0250065 PMC8049255 33857248

[23] Al-Kuwari MG. Tobacco control in Qatar. *Middle East J Fam Med.* 2008;6(6):11–3

[24] Papaloukas O, Giannouli G, Papaevangelou V. Successes and challenges in varicella vaccine. *Ther Adv Vaccines.* 2014;2(2):39–55 10.1177/2051013613515621 PMC3991154 24757524

[25] Al Kaabi N, Al Olama FM, Al Qaseer M, Al Ubaidani I, Dinleyici EC, Hayajneh WA, et al. The clinical and economic burden of varicella in the Middle East: a systematic literature review. Hum Vaccin Immunother. 2020;16(1):21–32. doi: 10.1080/21645515.2019.1638726 PMC7012098 31373864

[26] Al-Tawfiq JA, Memish ZA. Middle East respiratory syndrome coronavirus and severe acute respiratory syndrome coronavirus. Semin Respir Crit Care Med. 2020;41(4):568–578. doi: 10.1055/s-0040-1709160 PMC7516363 32305045

[27] Farag E, Sikkema RS, Vinks T, Islam MM, Nour M, Al-Romaihi H, et al. Drivers of MERS-CoV emergence in Qatar. Viruses. 2018;11(1):22. doi: 10.3390/v11010022 PMC6356962 30602691

[28] Ministry of Development Planning and Statistics 2014. Multiple indicators cluster survey, State of Qatar, 2012. Doha. Ministry of Development Planning and Statistics.2014. [cited 2021 Apr 2]. Available from: https://www.psa.gov.qa/en/statistics/Surveys/MICS-2012-Eng.pdf

[29] Nasser A, Omer F, Al-Lenqawi F, Al-awwa R, Khan T, El-Heneidy A, et al. Predictors of continued breastfeeding at one year among women attending Primary Healthcare Centers in Qatar: a cross-sectional study. Nutrients. 2018;10:983. doi: 10.3390/nu10080983 PMC6115788 30060523

[30] Al-Kuwari MG, Abdulla M, Bakri AH. Understanding breastfeeding practices among women registered at Primary Health Care Corporation Health Centres in Qatar. J Family Med Prim Care Open Acc. 2020;4:143. doi: 10.29011/2688-7460.100043

[31] The World Bank. 2021. Labor force participation rate, female indictor. Washington DC. The World Bank. 2021. [cited 2021 Jul 3]. Available from https://data.worldbank.org/indicator/SL.TLF.CACT.FE.ZS?locations = QA

